# Supplementary material for: Structure-based virtual screening, molecular docking, and MD simulation studies: An in-silico approach for identifying potential MBL inhibitors
Source: PLoS One. 2025 Jul 31;20(7):e0324836. doi: 10.1371/journal.pone.0324836 (PMC12312920; doi:10.1371/journal.pone.0324836)
Supplement: S4 Table — (DOCX) [file pone.0324836.s004.docx]

**Table S1.** Top binding energies, normalized binding energies of the screened selected compounds along with control

| Top BE | Compound | Normalized | Top BE | Compound | Normalized | Top BE | Compound | Normalized | Top BE | Compound | Normalized |
| --- | --- | --- | --- | --- | --- | --- | --- | --- | --- | --- | --- |
| -8.4 | D751-0254 | -7.88889 | -7.6 | S720-2243 | -6.94445 | -7.5 | S720-2427 | -6.67777 | -6.8 | S596-0076 | -6.47778 |
| -8.8 | D751-0277 | -7.83334 | -7.7 | S596-0645 | -6.93333 | -7.7 | D751-0121 | -6.66667 | -6.9 | S721-0470 | -6.47778 |
| -8.3 | N115-0050 | -7.82222 | -7.5 | S720-0483 | -6.93333 | -7 | S720-1059 | -6.66667 | -7.2 | S721-0984 | -6.47778 |
| -8.4 | D751-0223 | -7.81111 | -7.5 | S720-2578 | -6.93333 | -7.3 | S720-0361 | -6.65556 | -7.1 | S721-1960 | -6.47778 |
| -8.4 | D751-0258 | -7.76667 | -7.4 | S904-0098 | -6.93333 | -7.2 | S904-1695 | -6.65556 | -7.8 | S740-0946 | -6.47778 |
| -8.2 | S596-0634 | -7.75555 | -7.3 | S625-0626 | -6.92223 | -7.1 | S943-0427 | -6.65555 | -7.1 | S904-0052 | -6.47778 |
| -8 | N121-1124 | -7.67778 | -7.5 | S625-0782 | -6.92222 | -7.1 | N124-0101 | -6.64444 | -6.9 | S904-0055 | -6.47778 |
| -8.5 | S624-0349 | -7.64444 | -7.3 | S721-0519 | -6.91111 | -7.1 | S578-0931 | -6.64444 | -7 | S904-0937 | -6.47778 |
| -8.2 | SB73-0531 | -7.63333 | -7.7 | S904-1800 | -6.91111 | -7.1 | S720-2608 | -6.64444 | -7.1 | S578-0880 | -6.46667 |
| -9 | D751-0141 | -7.62223 | -8.1 | N109-0056 | -6.9 | -6.9 | S720-2312 | -6.63334 | -7.5 | S596-0569 | -6.46667 |
| -8.2 | N118-0133 | -7.6 | -7.3 | S596-0008 | -6.9 | -7.1 | S577-0574 | -6.63333 | -6.9 | S596-0607 | -6.46667 |
| -8.1 | SB74-0449 | -7.52222 | -7.7 | S596-0060 | -6.88889 | -7 | S625-0797 | -6.63333 | -7.2 | SB29-0573 | -6.46667 |
| -8.1 | N115-0003 | -7.48889 | -7.3 | S721-0324 | -6.88889 | -7 | S720-2455 | -6.63333 | -7.5 | S596-0054 | -6.45556 |
| -7.9 | N118-0137 | -7.48889 | -7.4 | S904-1329 | -6.88889 | -7.2 | S720-2592 | -6.63333 | -6.9 | S740-0255 | -6.45556 |
| -8.3 | D751-0090 | -7.47778 | -7.6 | T956-0166 | -6.88889 | -7.5 | S740-1248 | -6.63333 | -6.9 | S904-1687 | -6.45556 |
| -7.7 | S596-0088 | -7.45556 | -7.2 | S943-0424 | -6.87778 | -7.9 | SB29-0557 | -6.63333 | -7.1 | SB18-0280 | -6.45555 |
| -7.7 | S596-0112 | -7.44444 | -7.4 | N118-0004 | -6.87777 | -7 | SB73-0885 | -6.63333 | -7 | SB73-0409 | -6.45555 |
| -7.8 | S720-2408 | -7.43333 | -7.4 | SB18-0240 | -6.87777 | -7 | S596-1304 | -6.62222 | -6.9 | N124-0044 | -6.44444 |
| -8.7 | D751-0140 | -7.42222 | -7.4 | S904-1841 | -6.86667 | -6.9 | S721-0979 | -6.62222 | -6.9 | S577-0605 | -6.44444 |
| -7.9 | S625-0582 | -7.42222 | -7.3 | CM4579-5819 | -6.86666 | -7.2 | S904-1693 | -6.62222 | -7 | S904-1423 | -6.44444 |
| -7.8 | S904-1864 | -7.42222 | -7.3 | S721-2090 | -6.86666 | -6.8 | T956-0115 | -6.62222 | -6.9 | S904-1455 | -6.44444 |
| -8.2 | S596-0617 | -7.41111 | -7.7 | D751-0170 | -6.85556 | -6.9 | S596-0529 | -6.61111 | -7.5 | S904-1267 | -6.43334 |
| -8 | S596-1289 | -7.38889 | -7.3 | D751-0218 | -6.85555 | -7.1 | S596-0581 | -6.61111 | -7.1 | S720-2381 | -6.43333 |
| -7.7 | S596-1012 | -7.36667 | -7.1 | S721-2031 | -6.85555 | -7.2 | S720-0969 | -6.61111 | -6.8 | S578-0872 | -6.42222 |
| -8.2 | SB73-0565 | -7.36667 | -7.5 | S596-0043 | -6.84445 | -7.3 | S596-0021 | -6.6 | -6.6 | S596-0626 | -6.42222 |
| -7.9 | D751-0168 | -7.36666 | -7.4 | S925-0098 | -6.84445 | -6.9 | S625-0287 | -6.6 | -6.6 | S720-0607 | -6.42222 |
| -8.1 | D751-0146 | -7.33333 | -7.3 | D751-0214 | -6.84444 | -6.8 | S720-2604 | -6.6 | -7 | S721-1034 | -6.42222 |
| -7.7 | S596-0776 | -7.33333 | -7.4 | S720-2590 | -6.82222 | -7.2 | N124-0046 | -6.58889 | -7 | S904-0013 | -6.42222 |
| -7.9 | D751-0260 | -7.32223 | -7.1 | S740-0140 | -6.82222 | -6.9 | S740-0939 | -6.58889 | -6.6 | S720-2591 | -6.41111 |
| -7.7 | D751-0169 | -7.28889 | -7.1 | S720-2279 | -6.81111 | -7.1 | S721-1046 | -6.57778 | -6.9 | S904-0022 | -6.41111 |
| -8.2 | D751-0165 | -7.26667 | -7.5 | S740-0606 | -6.81111 | -7.4 | S740-0130 | -6.57778 | -6.9 | SB18-0273 | -6.41111 |
| -8 | S596-1142 | -7.24445 | -7.4 | SB35-0014 | -6.81111 | -7 | S721-2193 | -6.56667 | -6.8 | S720-2273 | -6.4 |
| -7.8 | S625-0614 | -7.24444 | -7.8 | S596-0489 | -6.8 | -7.3 | S596-0009 | -6.56666 | -6.6 | S721-2199 | -6.4 |
| -7.7 | S721-0358 | -7.23333 | -7.4 | S925-0132 | -6.8 | -7 | S578-0873 | -6.55556 | -7 | S904-1428 | -6.4 |
| -7.8 | S720-2453 | -7.21111 | -7 | S720-2612 | -6.78889 | -7.1 | SA23-1368 | -6.55556 | -6.8 | S720-1076 | -6.38889 |
| -7.8 | S720-2576 | -7.21111 | -7 | S720-0318 | -6.77778 | -7.2 | SB18-1323 | -6.55556 | -6.9 | SB73-0869 | -6.38889 |
| -7.8 | D751-0089 | -7.17778 | -7.6 | S721-0355 | -6.77778 | -7 | T956-0193 | -6.55556 | -7 | SB73-0878 | -6.38889 |
| -7.5 | S596-0083 | -7.17778 | -6.9 | S721-0513 | -6.77778 | -7.6 | S721-1002 | -6.55555 | -6.7 | N124-0120 | -6.37778 |
| -7.8 | SB73-0530 | -7.17778 | -7.1 | S721-2045 | -6.77778 | -6.8 | S721-1654 | -6.55555 | -7.4 | S625-1730 | -6.37778 |
| -7.9 | D751-0205 | -7.17777 | -7.3 | S721-2087 | -6.77778 | -6.8 | S904-0121 | -6.55555 | -6.9 | S721-0564 | -6.37778 |
| -7.9 | S904-1803 | -7.17777 | -7.6 | S943-0432 | -6.77778 | -7.1 | S720-0542 | -6.54445 | -7.3 | S721-1190 | -6.37778 |
| -7.7 | D751-0098 | -7.16667 | -7.7 | D751-0172 | -6.76667 | -7 | S720-2428 | -6.54445 | -7 | S825-2241 | -6.37778 |
| -7.5 | S904-1270 | -7.15555 | -7.6 | S720-2284 | -6.75556 | -7 | SB73-0438 | -6.54445 | -7 | S904-1271 | -6.37778 |
| -7.6 | S904-1849 | -7.15555 | -7.2 | S904-1253 | -6.75556 | -6.8 | S904-1461 | -6.54444 | -7.5 | S740-1240 | -6.37777 |
| -8 | S625-0783 | -7.14445 | -7.1 | SB73-0824 | -6.75556 | -6.8 | N124-0053 | -6.53333 | -6.8 | SB18-1270 | -6.37777 |
| -8 | S720-2597 | -7.14445 | -7.4 | T956-0239 | -6.75556 | -7.1 | N124-0097 | -6.53333 | -7.1 | S625-1343 | -6.36667 |
| -7.5 | S596-0081 | -7.13333 | -7 | S625-1725 | -6.75555 | -6.9 | S578-0869 | -6.53333 | -6.7 | S720-2274 | -6.36667 |
| -7.5 | S720-2600 | -7.12223 | -7 | S904-1721 | -6.75555 | -7.6 | S720-0307 | -6.53333 | -6.8 | S721-0514 | -6.36667 |
| -7.4 | S596-0664 | -7.12222 | -7.2 | S904-0044 | -6.74444 | -6.8 | S721-0079 | -6.53333 | -7 | S904-1296 | -6.36667 |
| -7.4 | S721-0326 | -7.12222 | -7.4 | S904-1325 | -6.74444 | -7 | S721-1975 | -6.53333 | -7 | S904-1300 | -6.36667 |
| -7.7 | S625-0284 | -7.11111 | -8.1 | S904-1801 | -6.73334 | -7 | S904-0987 | -6.53333 | -6.9 | S740-0616 | -6.36666 |
| -7.8 | N106-0758 | -7.1 | -7.7 | S740-1137 | -6.73333 | -6.8 | S904-1479 | -6.53333 | -6.8 | S625-0798 | -6.35556 |
| -7.6 | S596-0097 | -7.1 | -7.1 | S904-0087 | -6.73333 | -6.7 | SB18-0252 | -6.53333 | -6.6 | S720-2446 | -6.35556 |
| -8 | S596-1271 | -7.1 | -7.4 | S720-2450 | -6.72222 | -7.4 | SB18-1265 | -6.53333 | -7.2 | S720-2456 | -6.35556 |
| -7.5 | S720-2440 | -7.1 | -7 | S720-2609 | -6.72222 | -6.8 | SB73-0796 | -6.53333 | -6.8 | S904-1846 | -6.35556 |
| -7.8 | S904-1307 | -7.1 | -7 | S721-2203 | -6.72222 | -6.9 | S740-0174 | -6.52223 | -6.7 | S720-1023 | -6.35555 |
| -7.3 | S625-0281 | -7.08889 | -7.1 | SB73-0792 | -6.72222 | -7.2 | N124-0139 | -6.52222 | -6.8 | S577-0471 | -6.34445 |
| -7.4 | S721-2208 | -7.08889 | -7.4 | S720-2566 | -6.71111 | -7.4 | S596-0010 | -6.52222 | -6.8 | S578-0909 | -6.34445 |
| -7.9 | D751-0143 | -7.06667 | -6.9 | S904-1235 | -6.71111 | -7 | S720-2416 | -6.52222 | -6.8 | S904-0049 | -6.34445 |
| -7.5 | S720-1048 | -7.06667 | -7.1 | S904-1427 | -6.71111 | -7 | S721-0958 | -6.52222 | -7.1 | S904-1008 | -6.34445 |
| -7.3 | N121-0700 | -7.04444 | -7.4 | T956-0179 | -6.71111 | -7.1 | S740-1330 | -6.52222 | -7 | S596-1470 | -6.34444 |
| -7.3 | S596-0007 | -7.04444 | -7.2 | S596-0892 | -6.7 | -6.8 | N124-0034 | -6.51111 | -7 | S904-0015 | -6.34444 |
| -8 | S596-1362 | -7.03334 | -7.8 | S596-1269 | -6.7 | -7.1 | S625-0790 | -6.51111 | -6.5 | S904-0116 | -6.34444 |
| -7.6 | S625-0721 | -7.03334 | -7.2 | S625-0313 | -6.7 | -6.7 | S720-0586 | -6.51111 | -6.9 | N124-0039 | -6.33333 |
| -7.7 | N121-1107 | -7.02222 | -7.1 | S625-0641 | -6.7 | -7 | S720-2601 | -6.5 | -7 | S577-0600 | -6.33333 |
| -7.3 | S625-0285 | -7.02222 | -7.4 | S625-0669 | -6.7 | -7.1 | S740-0192 | -6.5 | -6.5 | S720-2519 | -6.33333 |
| -7.4 | S720-2509 | -7 | -7.1 | S720-0484 | -6.7 | -7.3 | S904-1349 | -6.5 | -7.3 | S740-0183 | -6.33333 |
| -7.5 | S943-0433 | -7 | -7.4 | S904-1273 | -6.7 | -6.7 | S904-1394 | -6.5 | -6.9 | S740-0924 | -6.33333 |
| -7.3 | N121-1071 | -6.98889 | -7 | S970-0471 | -6.7 | -7.1 | S904-1422 | -6.5 | -7 | S904-0075 | -6.33333 |
| -7.9 | S740-0945 | -6.98889 | -7.3 | T956-0129 | -6.7 | -7.2 | SB18-0220 | -6.5 | -6.8 | S721-0486 | -6.32223 |
| -7.3 | S720-2571 | -6.97778 | -7.1 | S721-0026 | -6.68889 | -7 | S596-0016 | -6.48889 | -6.8 | S721-2188 | -6.32223 |
| -7.4 | S720-2188 | -6.96666 | -7.2 | S578-0438 | -6.67778 | -7.4 | S740-1140 | -6.48889 | -6.6 | S740-0182 | -6.32222 |
| -7.7 | S596-0037 | -6.95556 | -7.6 | S721-0978 | -6.67778 | -6.9 | S904-1720 | -6.48889 | -6.9 | S904-1346 | -6.32222 |
| -7.2 | S720-2148 | -6.95555 | -7.1 | S904-1840 | -6.67778 | -6.9 | T956-0112 | -6.48889 | -7.2 | S904-1500 | -6.32222 |
| -8 | S740-0156 | -6.95555 |  |  |  | -7.3 | S578-0771 | -6.47778 | -7 | S943-0430 | -6.32222 |
|  |  |  |  |  |  |  |  |  | -6.8 | control_ORV | -6.31111 |
