# Supplementary figures and images for: Structure-based virtual screening, molecular docking, and MD simulation studies: An in-silico approach for identifying potential MBL inhibitors
Source: PLoS One. 2025 Jul 31;20(7):e0324836. doi: 10.1371/journal.pone.0324836 (PMC12312920; doi:10.1371/journal.pone.0324836)

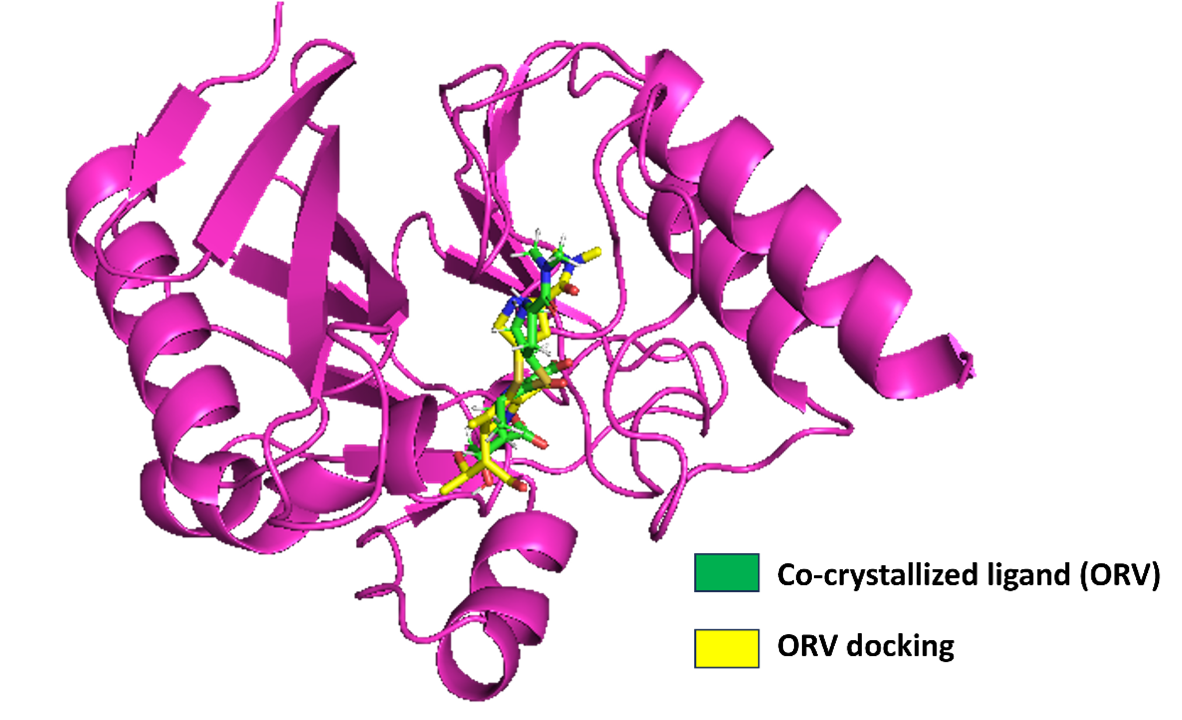

Supplement: S1 Fig — Legend: The protein backbone is shown in magenta cartoon representation. The original ligand conformation from the crystal structure is shown in green, while the re-docked pose is displayed in yellow. (PNG) [file pone.0324836.s005.png]

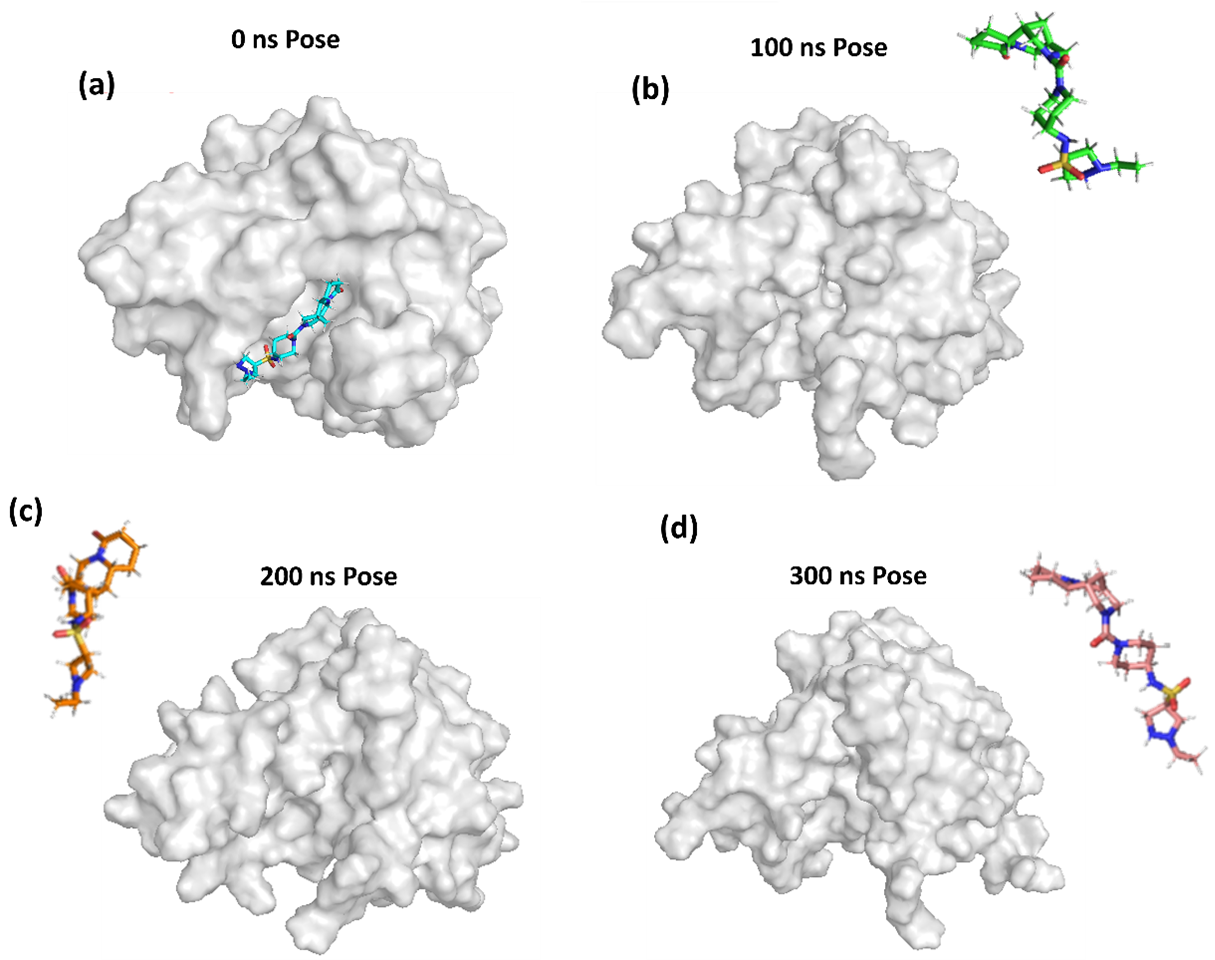

Supplement: S2 Fig — (PNG) [file pone.0324836.s006.png]

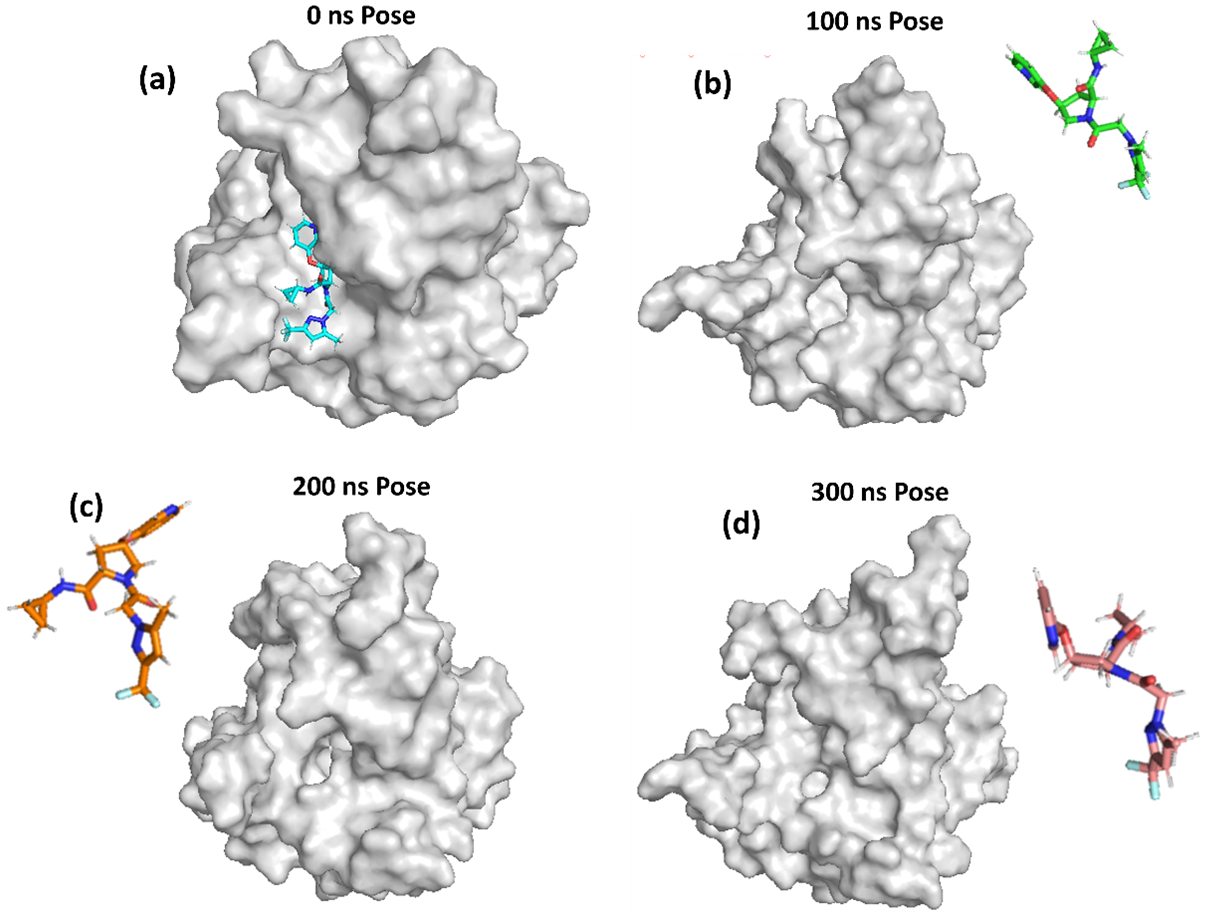

Supplement: S3 Fig — (PNG) [file pone.0324836.s007.png]

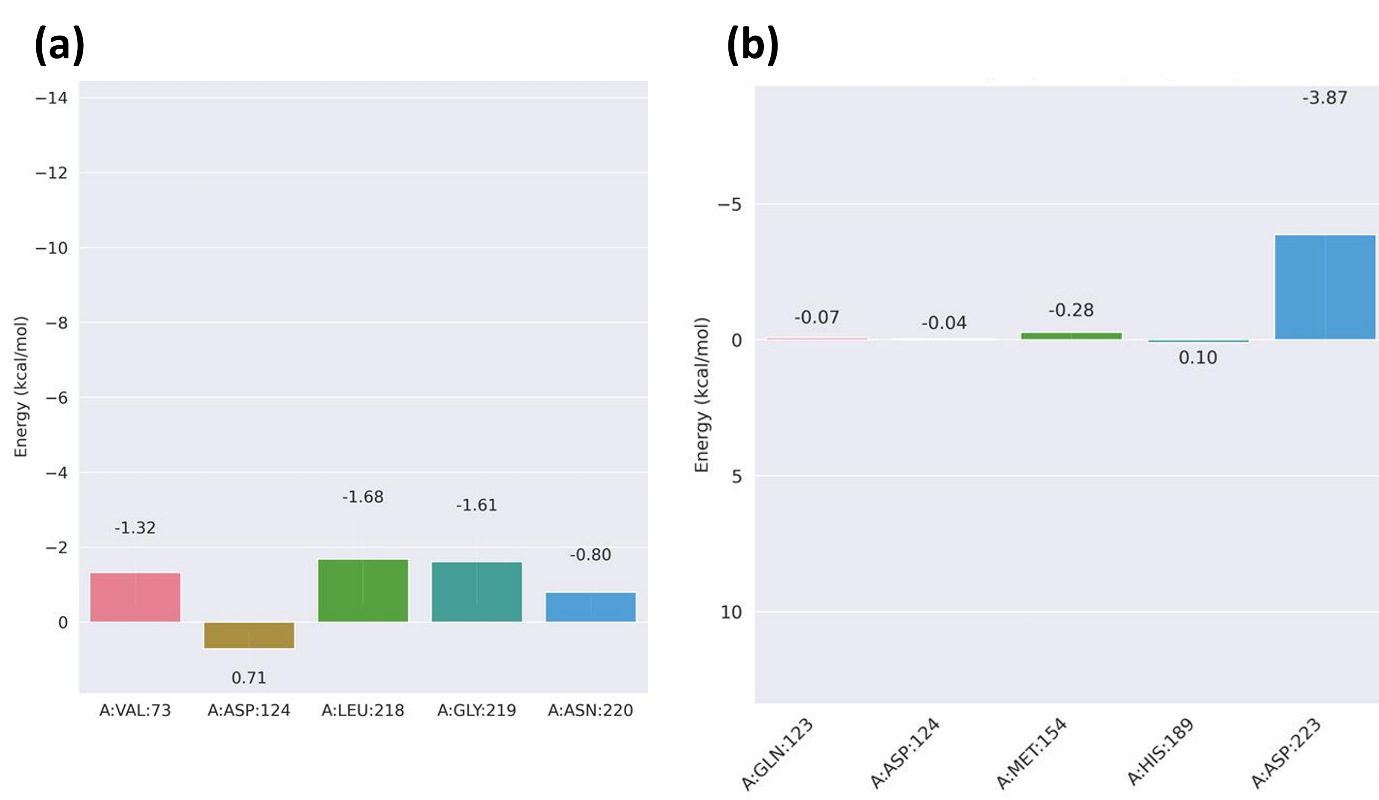

Supplement: S4 Fig — (PNG) [file pone.0324836.s008.png]
