# Supplementary material for: Structure-based virtual screening, molecular docking, and MD simulation studies: An in-silico approach for identifying potential MBL inhibitors
Source: PLoS One. 2025 Jul 31;20(7):e0324836. doi: 10.1371/journal.pone.0324836 (PMC12312920; doi:10.1371/journal.pone.0324836)
Supplement: Attachment — Submitted filename: PONE-D-25-11337__REVIEW.pdf [file pone.0324836.s013.pdf]

PLOS ONE REVIEWER GUIDELINES

- What are the main claims of the paper and how significant are they for the discipline?

  I think they are moderately significant.

- Are the claims properly placed in the context of the previous literature? Have the authors treated the literature fairly?

  Yes, fairly well.

- Do the data and analyses fully support the claims? If not, what other evidence is required?

  Yes, data supports claim.

- *PLOS ONE* encourages authors to publish detailed protocols and algorithms as supporting information online. Do any particular methods used in the manuscript warrant such treatment? If a protocol is already provided, for example for a randomized controlled trial, are there any important deviations from it? If so, have the authors explained adequately why the deviations occurred?

  Adequately, but more information / request is provided in notes below.

- If the paper is considered unsuitable for publication in its present form, does the study itself show sufficient potential that the authors should be encouraged to resubmit a revised version?

  Yes, the paper is good for publication upon minor revision detailed below.

- Are original data deposited in appropriate repositories and accession/version numbers provided for genes, proteins, mutants, diseases, etc.?

  Original data are cited. More requests in notes below.

- Does the study conform to any relevant guidelines such as CONSORT, MIAME, QUORUM, STROBE, and the Fort Lauderdale agreement?

  Does not seem applicable here.

- Are details of the methodology sufficient to allow the experiments to be reproduced?

  Nearly so; suggestions for improvement provided in notes below.

- Is any software created by the authors freely available?

Does not seem applicable here.

- Is the manuscript well organized and written clearly enough to be accessible to non-specialists?

  Nearly so; suggestions for improvement provided in notes below.

- Is it your opinion that this manuscript contains an NIH-defined experiment of Dual Use concern?

  Not at this time.

MY COMMENTS / RECOMMENDATIONS / REQUESTS

- For the sake of readability, I ask that the Authors rephrase the first sentence of the abstract to clarify that antibiotic resistance is what is being proliferated (if that is the intent of the Authors), and not that the very identification of NDM-1 causes a proliferation of antibiotic resistance. Also, I ask that all abbreviations are defined throughout the manuscript, including in the abstract (examples include ML, CHARMM36, CGneFF, LINCS, NVT, NPT, PC, and PCA).
- As with all abbreviations, I recommend writing techniques like "quantitative structure-activity relationship" (QSAR) and "molecular dynamics" (MD) in lower case.
- Line 67 – "Expanding upon this basis, a recent work employed a multi-step virtual screening method for the identification of non-β-lactam inhibitors against NDM-1." Lacking citation to the "recent work".
- Line 76-81 – "The objective of this investigation was to employ computational methods to comprehensively investigate and identify natural product compounds that exhibit inhibitory activity against NDM-1." I recommend rewording as "identify and investigate", in consistence with the flow of the story. Also, I recommend caution on the use of past tense to describe the objective of the study. Finally, there seem to be a duplicate statement of objective, I therefore recommend making that section more succinct and thus avoid potential confusion.
- Line 83 – I recommend Authors should not make the claim of "strong interactions" until the results have been presented (and discussed).
- Line 84 – For clarity and readability, and to avoid bogus claims, I recommend the language of "identified 'an inhibitor'", not 'the inhibitor'
- Line 141 – I recommend that Authors should refrain from hyphenating or abbreviating molecular dynamics simulations; it is not conventional to do so. Same issue in line 169 with free energy landscape.
- Line 149 – For readability, Authors should consider rewording "for the neutralization its charge"
- Line 152 – The "raised to" statement did not initially clarify a reference temperature. I strongly recommend that Authors make clear and simple the procedural steps involved in all analyses.
- Line 152 – "In addition, the entire system was raised to a temperature of 310 K using a timestep of 2 fs for a simulation time of 100 ps in the NVT ensemble and pressure (NPT) for a duration of 1 ns each at 310 K and 1 atmosphere."
  The statement seem to lack clarity and contains too many contradictory contents; some thoughts: what is the reference temperature of the post-minimization simulation, was there an annealing step, how long were the isothermal or annealing

steps (what is the distinction between the 100 ps and 1 ns mentioned), what was the timestep for each? These were not immediately clear upon first-time reading, hence the need to detail all procedures in plain language as much as possible.

- Line 155 – Authors should use conventional language; "production" rather than "manufacturing" for the data collection phase of the molecular dynamics simulations
- Line 156 – To enhance clarity and readability, Authors should consider rewording "Velocity scaling employed to ensure a constant environment of simulation". The statement should also be clarified to be pertaining to system temperature and not merely 'environment of simulation'.
- Line 159 – "The hydrogen bonding patterns within the protein-ligand complex were examined utilizing GROMACS' internal tools in order to gain a more comprehensive understanding of the dynamic interactions" – Authors should mention the specific GROMACS tool being used here, and describe its implementation. Also, Authors should stay consistent in representing the GROMACS software in uppercase—no need to provide full meaning (line 142).
- Line 176 – To enhance readability, Authors should consider placing a comma after "*kB*"
- Line 197 – To enhance readability, Authors should consider replacing "The that" with "The"
- Line 210 – Authors should consider supplying the appropriate citation to PyMol (and to every software being utilized for the study—as was done for GROMACS)
- Authors should consider including the unit of the distance column in Table 2.
- Section 3.4: RMSD – To strengthen claim, I recommend that the Authors make a short, informational comment (with citation(s)) on the potential effects of the protein instability that has been induced by ligand interactions, thus placing that protein stability information in proper context.
- I recommend that the Authors include the molecular structures of all four ligands (the control as well as the three identified) somewhere appropriate in the manuscript main. Also, was the control molecule the same for all analysis performed?
- Line 409 – Could the Authors make comments or provide citations to justify that "1-2 hydrogen bonds" is sufficient for **S904-0022** to be deemed relevant for further analyses including *in-vitro*.
